# Bacteriostatic and Cytotoxic Properties of Composite Material Based on ZnO Nanoparticles in PLGA Obtained by Low Temperature Method

**DOI:** 10.3390/polym14010049

**Published:** 2021-12-23

**Authors:** Dmitriy E. Burmistrov, Alexander V. Simakin, Veronika V. Smirnova, Oleg V. Uvarov, Petr I. Ivashkin, Roman N. Kucherov, Vladimir E. Ivanov, Vadim I. Bruskov, Mihail A. Sevostyanov, Alexander S. Baikin, Valery A. Kozlov, Maksim B. Rebezov, Anastasia A. Semenova, Andrey B. Lisitsyn, Maria V. Vedunova, Sergey V. Gudkov

**Affiliations:** 1 Prokhorov General Physics Institute of the Russian Academy of Sciences, 38 Vavilova St., 119991 Moscow, Russia; dmitriiburmistroff@gmail.com (D.E.B.); avsimakin@gmail.com (A.V.S.); veronausckova@mail.ru (V.V.S.); uvarov@kapella.gpi.ru (O.V.U.); ivashkin@kapella.gpi.ru (P.I.I.); rn.kucherov@gmail.com (R.N.K.); iwe88@rambler.ru (V.E.I.); rebezov@ya.ru (M.B.R.); 2 Moscow Engineering Physics Institute, National Research Nuclear University MEPhI, Kashirskoe Highway 31, 115409 Moscow, Russia; 3 Institute of Theoretical and Experimental Biophysics of the Russian Academy of Sciences, 3 Institutskaya St., 142290 Pushchino, Russia; bruskov_vi@rambler.ru; 4 A. A. Baikov Institute of Metallurgy and Materials Science of the Russian Academy of Sciences, Leninsky Prospect 49, 119991 Moscow, Russia; cmakp@mail.ru (M.A.S.); baikinas@mail.ru (A.S.B.); 5 Faculty of Fundamental Sciences, Bauman Moscow State Technical University, Vtoraya Baumanskaya Ul. 5, 105005 Moscow, Russia; v.kozlov@hotmail.com; 6 V. M. Gorbatov Federal Research Center for Food Systems of the Russian Academy of Sciences, 109316 Moscow, Russia; a.semenova@fncps.ru (A.A.S.); info@fncps.ru (A.B.L.); 7 Institute of Biology and Biomedicine, Lobachevsky State University of Nizhni Novgorod, 23 Prospekt Gagarina, 603950 Nizhny Novgorod, Russia; mvedunova@yandex.ru

**Keywords:** zinc oxide, nanoparticles, poly(lactic-co-glycolic acid), PLGA, composite, biocompatibility, antibacterial, cytotoxicity

## Abstract

A low-temperature technology was developed for producing a nanocomposite based on poly (lactic-co-glycolic acid) and zinc oxide nanoparticles (ZnO-NPs), synthesized by laser ablation. Nanocomposites were created containing 0.001, 0.01, and 0.1% of zinc oxide nanoparticles with rod-like morphology and a size of 40–70 nm. The surface of the films from the obtained nanomaterial was uniform, without significant defects. Clustering of ZnO-NPs in the PLGA matrix was noted, which increased with an increase in the concentration of the dopant in the polymer. The resulting nanomaterial was capable of generating reactive oxygen species (ROS), such as hydrogen peroxide and hydroxyl radicals. The rate of ROS generation increased with an increase in the concentration of the dopant. It was shown that the synthesized nanocomposite promotes the formation of long-lived reactive protein species, and is also the reason for the appearance of a key biomarker of oxidative stress, 8-oxoguanine, in DNA. The intensity of the process increased with an increase in the concentration of nanoparticles in the matrix. It was found that the nanocomposite exhibits significant bacteriostatic properties, the severity of which depends on the concentration of nanoparticles. In particular, on the surface of the PLGA–ZnO-NPs composite film containing 0.001% nanoparticles, the number of bacterial cells was 50% lower than that of pure PLGA. The surface of the composite is non-toxic to eukaryotic cells and does not interfere with their adhesion, growth, and division. Due to its low cytotoxicity and bacteriostatic properties, this nanocomposite can be used as coatings for packaging in the food industry, additives for textiles, and also as a material for biomedicine.

## 1. Introduction

The use of metal and metal oxides nanoparticles (NPs) as antibacterial agents is of great interest [1,2,3]. There are a large number of studies that have demonstrated a clear antibacterial effect of zinc oxide nanoparticles (ZnO-NPs). These nanoparticles are widely used for sensors [4] and solar panels [5], as well as in the cosmetic industry (sunscreens) [6]. Furthermore, ZnO-NPs find application in bioimaging due to their chemiluminescent properties [7]. ZnO-NPs exhibit cytostatic activity against cancer cells [8], as well as antifungal [9] and antimicrobial activity [2]. The main advantages of ZnO-NPs, in comparison with other nanomaterials, are their high antibacterial efficiency at low concentrations (0.16–5.00 mmol/L), activity against a wide range of bacterial strains, relatively low cost, and ease of synthesis [10]. Nanostructured ZnO can have various morphological forms and properties, depending on the synthesis routes and conditions. ZnO nanoparticles are synthesized by the sol–gel method [11], laser ablation [12], as well as simple chemical deposition [13]. The most common morphological forms of ZnO-NPs are nanospheres, nanorods, and nanoflowers [2].

The main mechanism of the antibacterial action of zinc oxide nanoparticles is the formation of reactive oxygen species (ROS) during the photocatalytic reaction. The formed ROS, mainly hydroxyl radicals (^•^OH), superoxide anions (O_2_^•−^), and hydrogen peroxide (H_2_O_2_), cause oxidative stress and damage to cell biopolymers [14,15]. One of the promising strategies for increasing the antibacterial properties and controlling the biocompatibility of NPs is the creation of polymer-NPs composites. The addition of NPs to the polymer matrix improves the performance of both the polymer backbone and the added nanomaterials. In particular, in composite materials, the stability of nanoparticles increases. Furthermore, the biological and mechanical properties of the polymer used are improved in the form of an increase in its biocompatibility, biodegradability, antibacterial activity, as well as strength and heat resistance. There is a wide range of polymers used as matrices for composites. These include compounds such as chitosan [16,17], gelatin [18], polyvinyl alcohol (PVA) [19,20,21], polylactate (PLA) [22], polypropylene [15], borosiloxane [23,24], and PLGA (poly (lactic-co-glycolic acid)) [25,26].

PLGA is a polymer often used as an organic component of composite scaffolds due to its good biocompatibility [27]. In the body, PLGA is broken down into polyglycolic acid (PGA) and polylactic acid (PLA). The successful creation of composites, based on PLGA-containing growth factors [28,29] and nanoparticles of metals and metal oxides [27,30,31,32], was noted. PLGA is also widely studied for drug delivery and tissue regeneration [33,34,35]. Along with good biocompatibility, PLGA helps to adjust the biodegradation time of the composite in the recipient’s body by changing the ratio of lactide and glycolide monomers in its composition [32,36]. The creation of PLGA-ZnO-NPs composites is of great interest, since the use of a PLGA matrix will control the release of ZnO-NPs in the body, thereby providing controlled cytotoxicity and optimal antibacterial properties against specific bacterial strains. In this study, we synthesized a PLGA–ZnO composite. The resulting composite was characterized using transmission electron microscopy (TEM), modulation interference microscopy (MIM), dynamic light scattering (DLS), and atomic force microscopy (AFM) methods. We also investigated the bacteriostatic and cytotoxic properties of synthesized composites with various concentrations of ZnO-NPs.

## 2. Materials and Methods

### 2.1. Apparatus

Zetasizer Ultra Red Label (Malvern Panalytical, Malvern, UK) was used for DLS analysis of colloidal solutions of zinc oxide nanoparticles. A Libra 200 FE HR transmission electron microscope (Carl Zeiss, Jena, Germany), in combination with a JED-2300 energy-dispersive X-ray spectrometer, was used to assess the morphology of the resulting nanoparticles. We used the CPS 24,000 disc analytical centrifuge (CPS Instruments, Prairieville, LA, USA) to determine the size distribution of nanoparticles. A double monochromator spectrometer Cintra 4040 (GBC Scientific Equipment, Braeside, Australia) was used to obtain the absorption spectrum of the obtained nanoparticles. A rheometer MCR 302e (Anton Paar, Graz, Austria) was used to measure the rheological characteristics of the obtained composite materials. A highly sensitive chemiluminometer, Biotox-7A-USE (ANO Engineering Center—Ecology, Russia), was used to register and measure luminescence in determining the concentration of reactive oxygen species, as well as for studying long-lived reactive protein species. A flatbed photometer (Titertek Multiscan, Finland) was used for the ELISA. A differential scanning calorimeter DSC 3 Excellence (Mettler Toledo, Columbus, OH, USA) was used for the thermal characteristics assay. A JASCO 8300 spectrofluorometer (JASCO, Tokyo, Japan) was used to register 7-OH-CCA fluorescence. Inverted microscope Leica DMI6000 (Leica Microsystems, Wetzlar, Germany) was used for microscopic analysis of bacterial and animal cells. An ES-20 incubator shaker (Biosan, Riga, Latvia) was used for the cultivation of bacterial cultures. A drop spectrometer, UV5Nano Excellence (Mettler Toledo, Columbus, OH, USA), was used to measure the optical density of a bacterial medium containing bacteria. A CO_2_ incubator S-Bt Smart Biotherm incubator (Biosan, Riga, Latvia) was used to cultivate permanent cell lines in in vitro studies.

### 2.2. Materials

Coumarin-3-carboxylic acid (CCA) (Sigma, Burlington, MA, USA) was used to quantify the generated hydroxyl radicals in solution. 7-hydroxycoumarin-3-carboxylic acid (7-OH-CCA) (Sigma, Burlington, MA, USA) was used for calibration. Monoclonal antibodies specific to 8-oxoguanine (Lab. Isotope Studies, Pushchino, Russia) were used in ELISA analysis to identify 8-oxoguanine in DNA. An LB broth medium (Thermo Fishcher, Waltham, MA, USA) was used to cultivate *E. coli*. DMEM (Biolot, Sankt- Petersburg, Russia) containing 10% fetal bovine serum (Gibco, Carlsbad, CA, USA) and 30 μg/mL of gentamicin (PanEco, Moscow, Russia) was used as a medium for in vitro cultivation of the SH-SY5Y cell line. Fluorescent dyes, Hoechst 33342 (Sigma, Burlington, MA, USA) and propidium iodide (Sigma, Burlington, MA, USA), were used for in vivo staining of cell cultures when assessing viability.

### 2.3. Methods

#### 2.3.1. Preparation and Characterization of Zinc Oxide Nanoparticles

Laser ablation in liquid was used to synthesize zinc oxide nanoparticles. A pulsed fiber ytterbium-doped laser was used. The following parameters of the laser were used: wavelength—1064 nm, pulse duration—4–200 ns; pulse repetition rate—20 kHz; average power—up to 20 W; and pulse energy—1 mJ. Deionized water (V = 10 mL) was used as a working fluid. A chemically pure zinc sample was used as a target and was immersed in the working fluid. The liquid layer on the target was about 1 mm. The irradiation time ranged from 5 to 20 min. A detailed description of the setup for generating nanoparticles by laser ablation can be found in Zhilnikova et al., [37]. The Zetasizer Ultra Red Label (Malvern Panalytical, Malvern, UK) was used to determine the hydrodynamic diameter of the resulting nanoparticles as well as to obtain the zeta potential distribution. A more detailed description of the features of recording these parameters was described by us earlier [38]. Furthermore, a CPS 24,000 disc analytical centrifuge (CPS Instruments, Prairieville, LA, USA) was used to evaluate the diameter of the resulting nanoparticles. The morphological features of nanoparticles (shape, topology), as well as the elemental composition of nanoparticles, were studied using a Libra 200 FE HR transmission electron microscope (Carl Zeiss, Jena, Germany) in combination with a JED-2300 energy-dispersive X-ray spectrometer. To assess the morphological features of the surface of the obtained composite films, we used an interference-modulation microscope MIM-321. In addition, to confirm the composition of the obtained nanoparticles, the spectrum of aqueous NP colloids was recorded using a Cintra 4040 (GBC Scientific Equipment, Braeside, Australia). Earlier, we described in detail the features of recording the spectrum of aqueous colloids of nanoparticles [39].

#### 2.3.2. Composite Fabrication, Production of Plates from Composite Material, Rheological Properties

The low-temperature technology developed by us earlier was used to obtain a PLGA–ZnO-NPs composite material [40]. The resulting composite material was heated to 40 °C and then rolled through rolls. As a result, a composite film with a thickness of about 1000 μm was obtained. From the resulting film, they were then cut into rectangular samples with a size of 20 mm × 15 mm. A modular compact rheometer MCR 302e (Anton Paar, Graz, Austria) was used to measure the rheological characteristics of the obtained composite materials. To describe the non-Newtonian behavior of systems, we applied the approach proposed by Chausov et al., where multiparameter rheological equations were used in a wide range of shear rates [23].

#### 2.3.3. Hydrogen Peroxide Concentration Measurement

The concentration of the formed hydrogen peroxide in aqueous solutions was carried out using the highly sensitive chemiluminescence method with luminol-p-iodophenol-horseradish peroxidase system [23]. A highly sensitive chemiluminometer was used to register and measure luminescence Biotox-7A-USE (ANO Engineering Center—Ecology, Moscow, Russia). The calibration and registration procedure are described in detail in a number of our other works [41,42]. The sensitivity of this method made it possible to determine H_2_O_2_ at a concentration of <1 nM [43].

#### 2.3.4. Hydroxyl Radicals Concentration Measurement

To quantify the content of hydroxyl radicals in aqueous solutions, a reaction with coumarin-3-carboxylic acid (CCA) (Sigma, Burlington, MA, USA) was used. During the hydroxylation reaction, 7-hydroxycoumarin-3-carboxylic acid (7-OH-CCA) was formed, a convenient fluorescent probe for recording the concentration of OH radicals [44]. A JASCO 8300 spectrofluorometer (JASCO, Tokyo, Japan) was used to register 7-OH-CCA fluorescence) at λ_ex_ = 400 nm, λ_em_ = 450 nm. Calibration was performed using commercial 7-OH-CCA (Sigma, Burlington, MA, USA) [45].

#### 2.3.5. Long-Lived Reactive Protein Species Concentration Measurement

The chemiluminescent method is an effective and sensitive method for the determination of free radical reactions. The interaction of radicals is accompanied by the release of energy in the form of emitted light quanta [46]. Chemiluminometer Biotox-7A (ANO “Engineering Center—Ecology”, Moscow, Russia) was used to study long-lived reactive forms of proteins by measuring the chemiluminescence of protein solutions with increasing temperature. The measurements were carried out in the dark at room temperature in 20-mL plastic polypropylene vials. Protein solutions that were not heated were used as controls. A more detailed description of the method was presented in the work of Sharapov et al. [47].

#### 2.3.6. Enzyme-Linked Immunosorbent Assay (ELISA)

To quantify 8-oxoguanine in DNA, a non-competitive enzyme-linked immunosorbent assay (ELISA) was used using monoclonal antibodies specific to 8-oxoguanine. The optical density of the samples was measured with a flatbed photometer (Titertek Multiscan, Vantaa, Finland) at λ = 405 nm. The method was described in more detail earlier [48].

#### 2.3.7. Thermal Characteristics Assay

A thermal characteristics assay was carried out by differential scanning calorimetry with DSC 3 Excellence (Mettler Toledo, Columbus, OH, USA). Thermograms in the heating and cooling modes were constructed to assess thermal characteristics. The temperatures of glass transition (T_g_) and heat capacity change (ΔC_p_) were also evaluated at different dopant concentrations.

#### 2.3.8. Bacteriostatic Activity Assay

The bacteriostatic activity of the obtained PLGA–ZnO-NPs composites was evaluated against Gram-negative bacteria *Escherichia coli*. Samples of films 15 mm × 15 mm in size were preliminarily sterilized by soaking in 70% ethanol solution for 30 min. Then, the film was put on a sterile hoop, on which an LB broth (Thermo Fishcher, Waltham, MA, USA) culture medium with a known CFU number was then placed. The resulting construct was placed in an ES-20 incubator shaker (Biosan, Riga, Latvia) and cultured at 37 °C, approximately 60 rpm for 24 h. Using microscopy and the previously developed algorithm for determining optically dense objects in the frame, the concentration of bacterial cells was estimated during the cultivation time. At the end of the experiment, the structure was dismantled and the concentration of bacteria was estimated using a drop spectrometer UV5Nano Excellence (Mettler Toledo, Columbus, OH, USA) [49].

#### 2.3.9. Citotoxicity Study

The obtained samples of the PLGA–ZnO-NPs composite films were evaluated for their cytotoxicity in vitro against the permanent cell line of human neuroblastoma SH-SY5Y. These cells are a good model for studying the development and differentiation of cells in vitro. A feature of this cell line is the possibility of their growth both in a monolayer and in the form of aggregates in the volume of the medium [50]. DMEM (Biolot, Sankt- Petersburg, Russia) containing 10% fetal bovine serum (Gibco, Carlsbad, CA, USA) and 30 μg/mL of gentamicin (PanEco, Moscow, Russia) were used as a culture medium. The cultivation was carried out in S-Bt Smart Biotherm CO_2_ incubator (Biosan, Riga, Latvia) at a temperature of 37 °C and 5% CO_2_.

Sterile 20 mm × 20 mm PLG /ZnO-NPs composite samples were placed in 35-mm Ø Petri dishes for each of the samples. A suspension of SH-SY5Y cells (104 cells/cm^2^, V = 3 mL) was placed on the surface of the samples. The cultivation time in vitro on the surface of the studied films was 72 h. Then, the viability of cell cultures was assessed by staining the cultures with fluorescent dyes Hoechst 33,342 (Sigma, Burlington, MA, USA) and propidium iodide (Sigma, Burlington, MA, USA) at concentrations of 2 μg/mL each. Hoechst 33,342 stains both living and non-viable cells. Propidium iodide stains non-viable cells with a damaged cytoplasmic membrane. This dye penetrates into living cells extremely slowly.

An imaging system based on a Leica DMI6000 (Leica Microsystems, Wetzlar, Germany) was used for microscopic analysis of cells on the surface of PLGA–ZnO-NPs composite samples. During the analysis, at least 500 cells were counted on the surfaces of the films [51]. A series of images were taken on a randomly selected field of the crop under consideration in transmitted light, with filters for Hoechst and propidium iodide. The number of non-viable cells, the density of the cell culture, the percentage of a cell-free surface area, and also the mitotic index were estimated as the main parameters that determine the growth and development of cells using the ImageJ software. Evaluation of the mitotic index of cells was carried out to analyze cell proliferation. Cells in a state of mitosis were identified by the distribution of chromatin stained with Hoechst 33342 (Sigma, Burlington, MA, USA), characteristic of prophase (P), metaphase (M), anaphase (A), and telophase (T). The mitotic index (MI) was calculated using the formula MI = (P + M + A + T)/N × 100%, where (P + M + A + T) is the number of cells at the prophase, metaphase, anaphase, and telophase stages, respectively. N is the total number of analyzed cells. We previously described this method in more detail [52].

#### 2.3.10. Statistic

The data were analyzed using GraphPad Prism eight and Origin software and were presented as means ± SEM. Data from at least three independent experiments were used for averaging.

## 3. Results & Discussion

**Figure 1:**
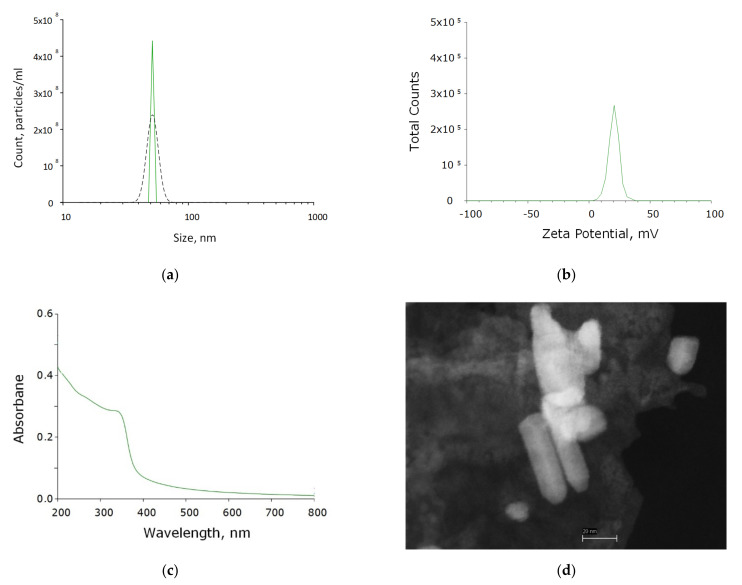
Physicochemical properties of ZnO-NPs. (**a**) Concentration (DLS, solid crimson line) and size distribution (CPS, black dashed line) of zinc oxide NPs. (**b**) Zeta potential of zinc oxide NPs. (**c**) Optical absorption of an aqueous colloidal solution of zinc oxide NPs. (**d**) TEM image of a group of zinc oxide NPs.

ZnO-NPs were synthesized by laser ablation in water. The concentration and hydrodynamic size of the resulting nanoparticles were determined using a DLS. The resulting colloidal solution contained ~450 million nanoparticles per ml. The average hydrodynamic nanoparticle diameter is 47 nm (Figure 1a). The size distribution of nanoparticles was monomodal with a distribution half-width of the order of 45–60 nm. Furthermore, the nanoparticle zeta potential profile was determined, which was distributed in the range from 6 to 40 nm with a maximum of 15 mV (Figure 1b). The composition of the obtained nanoparticles corresponding to zinc oxide was confirmed by absorption spectroscopy (Figure 1c). Morphological analysis by TEM showed the presence in the colloidal solution of nanoparticles with rod-like morphology, about 40–70 nm in length and Ø10 nm (Figure 1d).

**Figure 2:**
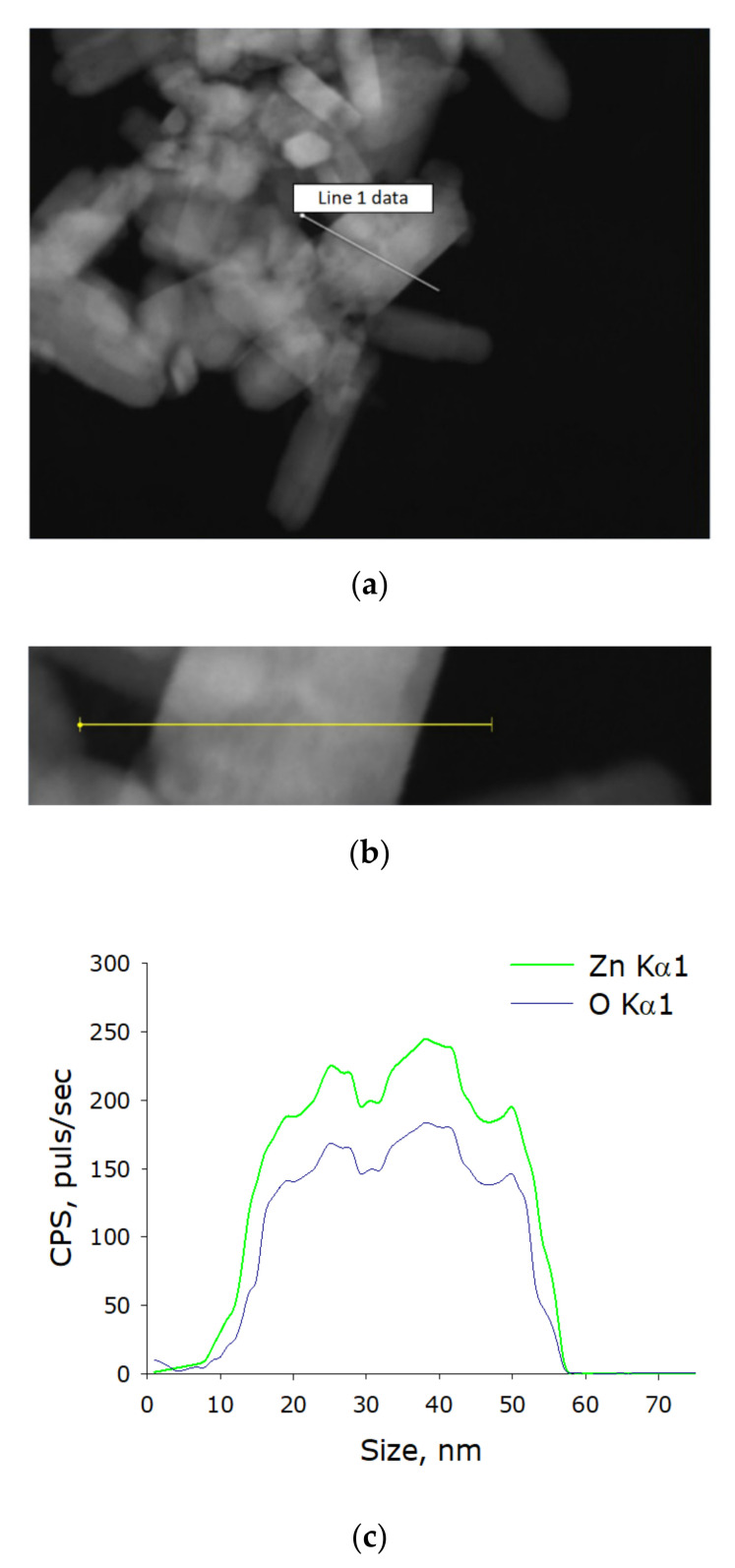
Elemental analysis of zinc oxide NPs. (**a**) TEM image of group of zinc oxide NPs, analysis section is indicated by line 1. (**b**) Enlarged measurement site. (**c**) Nanoparticle profile by Zn Kα1 and O Kα1.

Using energy-dispersive X-ray spectrometry, the elemental composition of the obtained nanoparticles was determined. The content of two elements in the NP composition was revealed: zinc and oxygen. The resulting nanoparticles contained ~90% ZnO and ~10% metallic zinc. Thus, it was found that the colloidal solution contained chemically pure zinc oxide NPs without impurities in their composition (Figure 2a–c).

**Figure 3:**
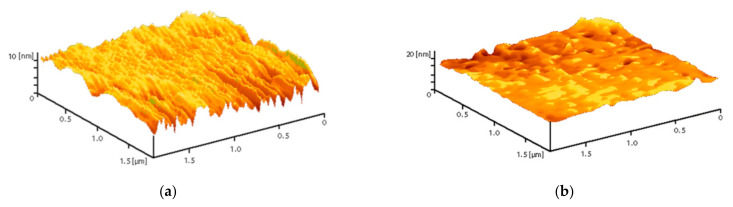
Reconstruction of the surface of a polymer and composites based on it, performed using an atomic force microscope in slow (**a**) and fast (**b**) scanning modes.

The PLGA–ZnO-NPs composite was fabricated using the low-temperature method that we developed earlier. The resulting composite material visually had a uniform and smooth surface. Using atomic force microscopy in two scanning modes (slow and fast scanning), it was found that the surface of the resulting composite was homogeneous and did not have cracks, breaks, and other artifacts (Figure 3).

**Figure 4:**
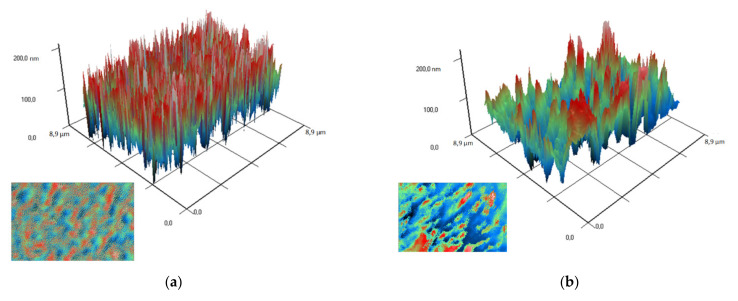

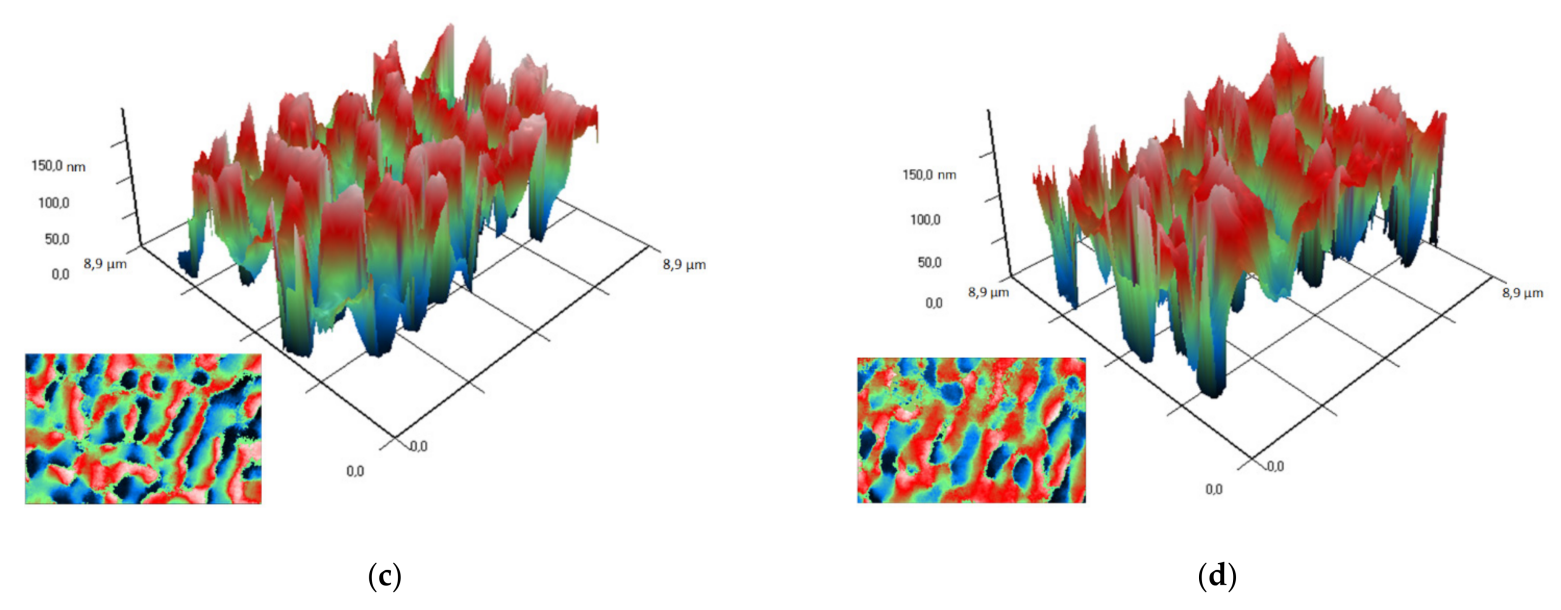
Images obtained on polymer without nanoparticles (**a**) and polymers with ZnO nanoparticles at a concentration of 0.001% (**b**), 0.01% (**c**) and 0.1% (**d**) using a modulation interference microscope. A 3D reconstruction of the surface profile of a polymer and composites based on it is presented. The X and Y axes show the actual size of the investigated surface in micrometers. The Z-axis shows the surface relief as a phase change expressed in nm. In the lower left corner of each figure is a top view (surface elevation map).

To study the arrangement of nanoparticles in the PLGA, the method of modulation interference microscopy (MIM) was used, which makes it possible to identify patterns in materials that differ in refractive index and other optical properties. The refractive index was determined at the wavelength of the laser microscope. The refractive index of the unmodified PLGA is 1.47 at 405 nm, and the refractive index of the zinc oxide is 2.02 at 405 nm. Thus, the refractive index of PLGA and zinc oxide nanoparticles differed by almost 0.5 units. It was found that the surface of PLGA without nanoparticles did not have a pronounced structuredness (Figure 4a). The addition of zinc oxide nanoparticles (0.001%) resulted in the formation of domains distinguished by the phase change in the laser radiation (Figure 4b). The fusion of the domains of nanoparticles with the formation of clusters, which were several mm in size, was noted with an increase in the concentration of ZnO-NPs to 0.1% (Figure 4c,d). Thus, the distribution of zinc oxide nanoparticles in the PLGA–ZnO-NPs composite was uneven.

It is possible to elucidate the type of interfacial polymer–nanoparticle interaction by the nature of changes in the thermodynamic parameters of the composite. This kind of research was carried out in the article [53]. However, the nanoparticles used in [53] were also inorganic, and the conclusions were confirmed by SAXS analysis. In our work, it was not possible to carry out SAXS structural analysis; therefore, it is difficult to draw unambiguous conclusions about the nature of the interaction between the dopant and the carrier medium.

**Figure 5:**
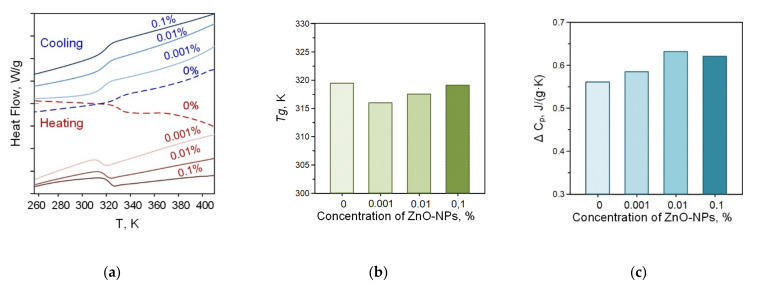
Thermograms of PLGA–ZnO in the heating and cooling mode (**a**); concentration dependences of changes in heat capacity (**b**); and glass transition temperature (**c**) of the PLGA–ZnO samples.

We also performed thermal analysis of the obtained PLGA–ZnO-NPs composites. Figure 5 shows thermograms of PLGA–ZnO-NPs samples obtained in heating and cooling mode. The numbers indicate different concentrations of ZnO-NPs in the composite. In the temperature range of 320–330 K, the glass transition process of the polymer is clearly visible, which is observed for all samples. The addition of ZnO-NPs to PLGA did not change the glass transition temperature. Based on the results of differential scanning calorimetry, the glass transition temperatures (Tg) and the change in heat capacity (ΔC_P_) of the samples in the study were determined, and the concentration dependences of which are shown in Figure 5b,c, respectively. The glass transition temperature is in the range of 317–319 K and corresponds to the literature data for pure PLGA. With increasing loading from 0.001% to 0.1%, the Tg increases due to restricted movement of the polymer chain by the incorporation of ZnO-NP in the polymer chain via electrostatic interaction. The ΔC_P_ values did not statistically change when nanoparticles were added to the PLGA formulation. There was a tendency towards an increase in the heat capacity of materials.

The composites used in this study have a mass fraction of zinc oxide nanoparticles of 0.001–0.1%. Such a small loading of the material does not significantly affect the thermodynamic properties of the carrier medium, since significant changes start from 1 wt% and above. Results on the effect of nanoparticles on the phase transitions of polymer composites can be found in [53,54,55,56,57]. Thus, our data correlate with the results obtained by other authors. Nevertheless, the material has significant bacteriostatic properties with the addition of ZnO nanoparticles. This is the improved properties of the composite material doped with nanoparticles.

**Figure 6:**
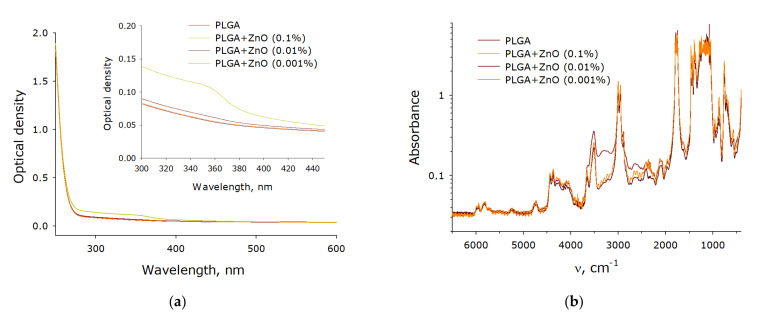
Spectral properties of a nanocomposite polymer material containing PLGA and ZnO nanoparticles. (**a**) Absorption UV-Vis spectrum of composite polymer materials with different filling with zinc oxide nanoparticles. (**b**) FTIR spectrum of composite polymer materials with different filling with zinc oxide nanoparticles.

The optical properties of composite polymer materials consisting of PLGA and ZnO nanoparticles have been investigated (Figure 6). It has been shown that the addition of nanoparticles at a concentration of 0.001% has almost no effect on the optical density of the polymer in the visible and part of the ultraviolet spectrum (Figure 6a). When zinc oxide nanoparticles at a concentration of 0.01% are added to the composite material, an insignificant increase in optical density is observed. The composite material containing 0.1% zinc oxide nanoparticles differed from the polymer not containing nanoparticles most significantly. In this case, a transition near 360 nm, characteristic of zinc oxide nanoparticles, was even observed. An FTIR study of a composite material containing zinc oxide nanoparticles was carried out (Figure 6b). In general, the spectra are extremely similar to each other. The only significant difference between a polymer containing no nanoparticles and polymers with nanoparticles is observed in the region from 3508 to 3005 cm^−1^. In a polymer without nanoparticles, a greater absorption is observed in this region, which can probably be associated with disordering and loosening of polymer chains in the composite material.

**Figure 7:**
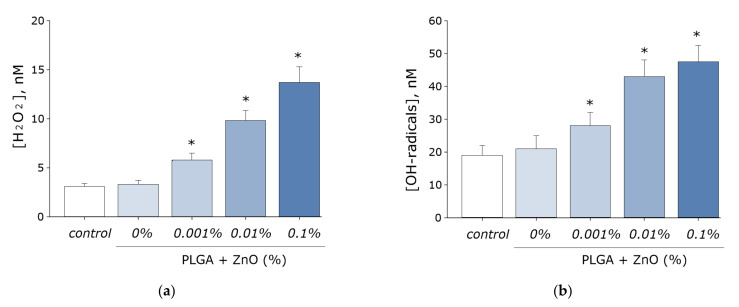
Effect of composite material containing PGLA and ZnO nanoparticles on the generation of reactive oxygen species: (**a**) Formation of hydrogen peroxide (2 h, 40 °C); (**b**) Generation of hydroxyl radicals (2 h, 80 °C); * indicate a significant difference at 5% level in comparison with the control (*p* < 0.05). Data are presented as mean values and standard errors of main.

It is known that zinc oxide promotes the formation of ROS during the photocatalytic reaction. The ability of the resulting PLGA–ZnO-NPs composite to generate ROS, such as hydrogen peroxide and hydroxyl radicals, was investigated. It was found that pure PLGA had no effect on the generation of ROS in aqueous solution. However, the PLGA–ZnO-NPs composite increased the rate of formation of hydrogen peroxide (Figure 7a) and hydroxyl radicals (Figure 7b) at all concentrations considered. An increase in the concentration of zinc oxide nanoparticles in the composition of the composite promoted an increase in the generation rate of the considered ROS. In particular, composites with the highest concentration of ZnO-NPs (0.1%) increased the formation of H_2_O_2_ by ~six times and OH-radicals by ~2.5 times.

**Figure 8:**
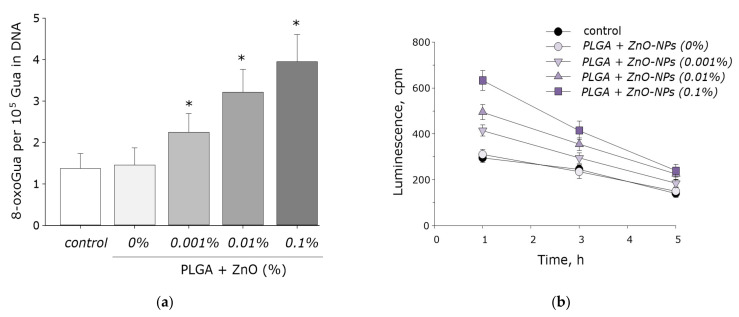
The effect PGLA/ZnO-NPs composite on the biomacromolecules damage formation: (**a**) Generation of 8-oxo-G in DNA in vitro (2 h, 45 °C); (**b**) Formation and dynamics of decomposition of long-lived reactive protein species (2 h, 40 °C); * indicate a significant difference at 5% level in comparison with the control (*p* < 0.05). Data are presented as mean values and standard errors of main.

It is known that ROS are capable of damaging biomolecules. The effect of the PLGA–ZnO composite on the formation of 8-oxoguanine in DNA in vitro was investigated. It was found that in vitro PLGA did not affect the rate of 8-oxoguanine formation in DNA. Upon doping ZnO nanoparticles into PLGA, an increase in the rate of 8-oxoguanine formation in DNA was observed in proportion to an increase in the concentration of ZnO-NPs. At a ZnO-NPs concentration of 0.001%, the rate of 8-oxoguanine formation in DNA increased 1.5 times; at a ZnO-NPs concentration of 0.01%, 2.3 times; and at a concentration of 0.1%, 2.8 times (Figure 8a).

We also investigated the effect of the PLGA–ZnO-NPs composite on the formation of long-lived reactive protein species. PLGA, which does not contain ZnO-NPs in its composition, did not affect the rate of degradation or generation of long-lived reactive protein species. Doping of the polymer with zinc oxide nanoparticles resulted in a statistically significant increase in the rate of formation of long-lived reactive protein species (Figure 8b). The addition of ZnO-NPs did not affect the half-life of the long-lived reactive protein species, which was on the order of 4–5 h.

**Figure 9:**
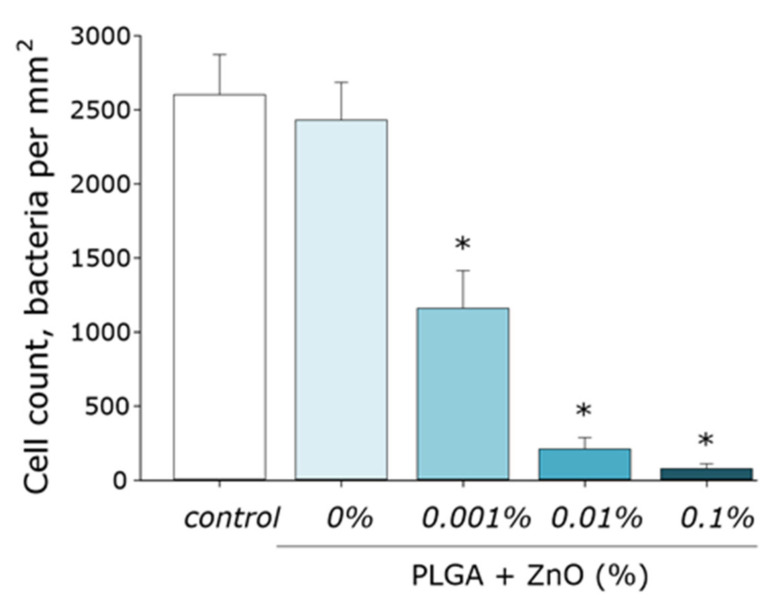
Influence of composite material based on PGLA and zinc oxide nanoparticles on the growth and development of *E. coli*. * indicate a significant difference at 5% level in comparison with the control (*p* < 0.05). Data are presented as mean values and standard errors of main.

The effect of the obtained PLGA–ZnO composites on the growth and development of *E. coli* bacterial cultures was studied. Pure PLGA, without the inclusion of ZnO-NPs, had no effect on bacterial cell growth. The addition of ZnO-NPs PLGA led to a sharp decrease in the number of bacterial cells on the surface of the composite. An increase in the concentration of ZnO-NPs led to an increase in the antibacterial effect. The number of cells on the surface of the composite with 0.001% ZnO-NPs decreased by two times; with 0.01% ZnO-NPs, 10 times. The PLGA–ZnO-NPs composite containing 0.1% ZnO-NPs had pronounced bacteriostatic properties (Figure 9).

**Figure 10:**
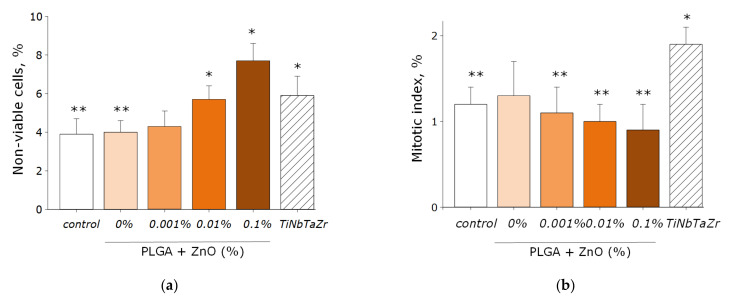

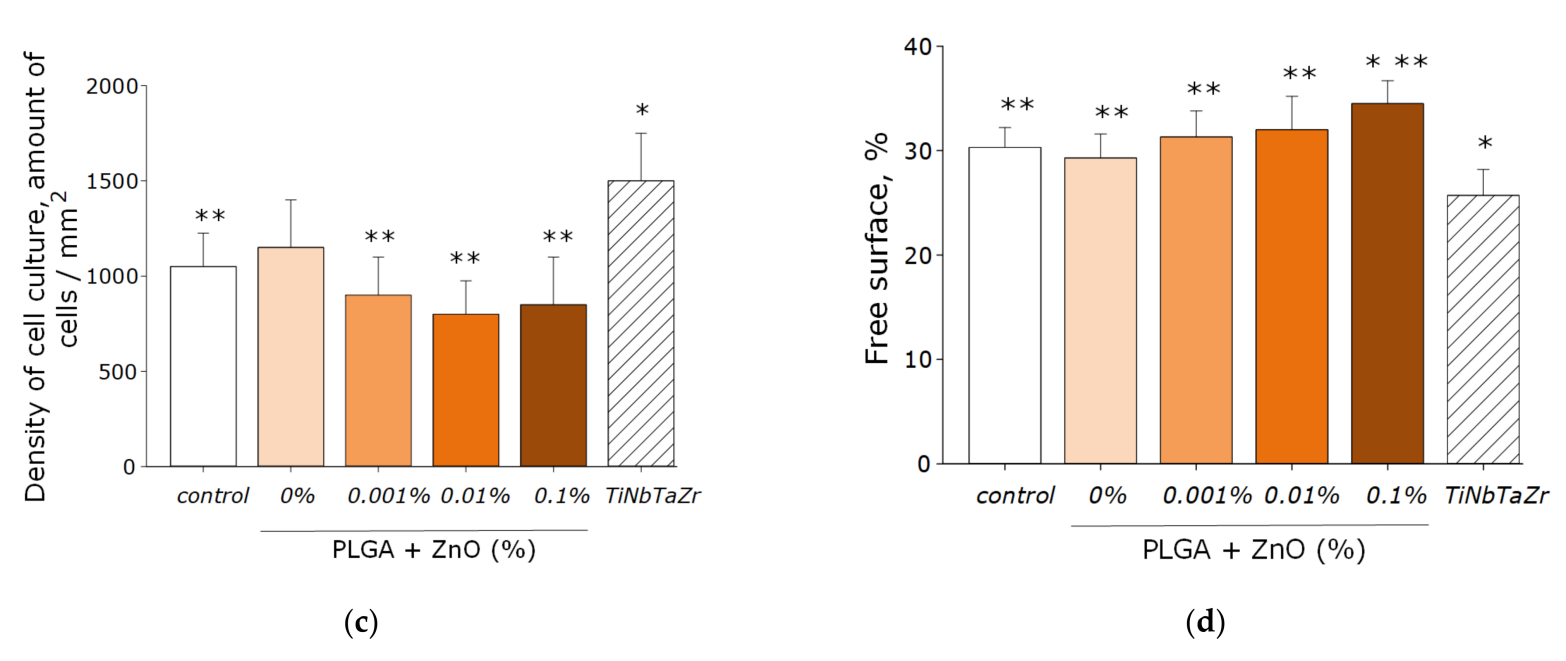
The effect of the PLGA/NP composite on the main characteristics of the growth and development of cell culture after 72-h cultivation: (**a**) Influence of composite material on the viability of cell culture; (**b**) Influence of composite material on the mitotic index of a cells; (**c**) Influence of composite material on the cell culture density; (**d**) Influence of composite material on the colonization rate of free surface by cells. * indicate a significant difference at 5% level in comparison with the control (*p* < 0.05). ** indicate a significant difference at 5% level in comparison with the TiNbTaZr group (*p* < 0.05). Data are presented as mean values and standard errors of main.

The effect of PLGA–ZnO-NPs on the growth and development of eukaryotic cell cultures was investigated (Figure 10a–d). It was found that the growth rate of cell cultures on the surface of the culture plastic (control group) was lower than on the surface of the TiNbTaZr medical alloy. This was manifested in a statistically significant decrease in the mitotic index and the cell density per unit area, as well as an increase in the area not occupied by cells in the control group. When PLGA, which does not contain ZnO-NPs, was used as a surface for cell adhesion, all the parameters considered did not change and were at the level of the ‘control’ group. About 4% of non-viable cells were observed, 1.3% of cells showed mitotic activity, the density of cultures was ~1200 cell/mm^2^, and ~30% of the polymer area was occupied by cells in 3 days. The formation of a single monolayer was not observed.

Doping PLGA with zinc oxide nanoparticles led to an increase in the percentage of nonviable cells. When using a PLGA-ZnO-NPs composite with a ZnO-NPs concentration of 0.1%, an increase in the number of non-viable cells, a decrease in the number of mitotic events and the density of cell cultures compared with the TiNbTaZr group, as well as an increase in the surface area not occupied by cells as compared to the control values with the TiNbTaZr group. Thus, it was shown that the PLGA-ZnO-NPs material with all investigated concentrations of nanoparticles is suitable for the adhesion, growth, and development of eukaryotic cells.

As is known, one of the main mechanisms of the antifungal, bacteriostatic, and bactericidal action of zinc oxide nanoparticles is the formation of reactive oxygen species during the photocatalytic reaction [15]. However, a high concentration of ROS leads to oxidative stress. Oxidation of biopolymers, in particular nucleic acids, leads to cell death. One of the main products of DNA oxidation is 8-oxoguanine DNA, which promotes the formation of mismatched nucleotides with adenine. As a result, the cellular repair system cannot cope with numerous damage to the DNA chain, which leads to the impossibility of matrix processes and cell death.

The PLGA-ZnO-NPs obtained by us increased the rate of ROS (H_2_O_2_ and OH-radicals) formation by several times. However, PLGA without NPs did not exhibit these properties. Moreover, the obtained nanocomposite promoted an increase in the formation of DNA oxidation products in vitro and a statistically significant increase in the rate of formation of long-lived active forms of proteins. The clear bacteriostatic effect of PLGA–ZnO composites on *E. coli* was also demonstrated. The PLGA matrix without the addition of nanoparticles had no effect on the growth and development of bacteria.

There are many relevant studies that have demonstrated the antibacterial effect of various polymers doped with zinc oxide nanoparticles (see Table 1). However, the high antibacterial potential of the composite is often accompanied by a cytotoxic effect on eukaryotic cells. The composite obtained by us based on PLGA and ZnO-NPs did not interfere with the adhesion of cells to its surface. The number of viable cells growing on the surface of the obtained composite corresponded to the values during growth on the surface of the TiNbTaZr medical alloy. The possibility of using polymer–ZnO-NPs composites as packaging [58,59,60], dressings [61,62], and wound-healing agents [63], as well as for the decomposition of wastewater [15], has been reported. An important property of PLGA as a matrix for the manufacture of composite materials is controlled biodegradability within the body [64,65]. Thus, the composite material developed by us PLGA–ZnO-NPs is promising for use in biomedical applications.

## 4. Conclusions

In the present study, a composite material based on PLGA and ZnO-NPs was synthesized and characterized. PLGA has a low manufacturing cost and is a “convenient” matrix for zinc oxide nanoparticles. It has been shown that such a composite material is capable of generating ROS and damaging biomacromolecules. “Empty” PLGA, without the addition of ZnO-NPs, does not exhibit bacteriostatic properties. The addition of ZnO-NPs to the PLGA composition, even at a minimal concentration (0.001%), was accompanied by a significant decrease in the number of bacterial cells per unit area. Thus, the resulting composite had a pronounced bacteriostatic effect, while it was not toxic to mammalian cells. The resulting composite based on PLGA and ZnO-NPs is of great interest for use as a packaging material in the food industry, additives for textiles, components for prostheses, and biomedical devices.

## Figures and Tables

**Table 1:** The main parameters of ZnO-NPs–polymer composites reported in other modern research.

No	Composition	D, nm	Bacterial Strains	Effect	MIC/MBC	Results	Ref.
1	Chitosan-ZnO-NPs composite	-	*E. coli*, *S. aureus 28*, *S. aureus ATTC*	BS *, BC	-	High antibacterial activity of the composite in comparison with pure chitosan	[58]
2	Nano-ZnO-high-density polyethylene (HDPE) composite films	~300	*S. aureus*, *E. coli*	BS	0.5 wt %	Nano-ZnO–HDPE composite films inhibited the antibacterial activity for *S. aureus* much more effectively than for *E. coli*	[66]
3	Cellulose-ZnO-NPs composite	20–40	*S. aureus*, *E. coli*	BS	-	Cellulose–ZnO have advantages compared to ZnO with the antibacterial activity of the composite better than ZnO	[67]
4	carboxymethyl chitosan (CMC) -carboxymethyl pullulan (CMP)–ZnO-NPs composites	~9	*S. aureus*, *E. coli*	BS	-	Antibacterial activity against both bacteria under investigation	[68]
5	chitosan (CS)/carboxymethyl cellulose (CMC)/ZnO-NPs composites	48–77	*S. aureus*, *E. coli*	BS	-	All of the composites have antibacterial activities and the highest antibacterial activity related to the synthesized composite without nano ZnO in *E. coli* medium culture and the best result achieved in prepared nano composite with nano ZnO in *S. aureus* medium	[59]
6	gelatin films with ZnO-NPs incorporation	80–100	*S. aureus*, *E. coli*	BS	-	The gelatin-based bio-nanocomposite films showed antibacterial properties against *Staphylococcus aureus*	[18]
7	poly vinyl alcohol (PVA) nanofibers incorporated with ZnO nanoparticles	~54	*S. aureus*, *E. coli*	BS	MIC: for *S. aureus* 250 μg/mL; for *E. coli* 62.5 μg/mL	PVA–ZnO composites exhibit antibacterial and wound healing properties	[63]
8	ZnO-NPs–cellulose nanocomposite	~14	*S. aureus*, *E. coli*	BS	-	Dispersing ZnO on the cellulose matrix improves the photocatalytic efficiency of ZnO; ZnO–CNC showed enhanced antibacterial activity against both *S. aureus* and *E. coli* compared to pure ZnO	[69]
9	PLA-PBAT-ZnO-NPs composite films	50–150	*E. coli*, *L. monocytogenes*	BS	-	Strong antibacterial activity against *E. coli* and *L. monocytogenes*	[60]
10	Polypropylene (PP) -ZnO-NPs nanocomposites	54–90	*E. coli*	BS	-	The PP–ZnO nanocomposites had better antibacterial properties than neat PP	[15]

* BS—bacteriostatic effect, BC—bactericidal effect.

## Data Availability

The data presented in this study are available on request from the first author.
